# Geopolitical Malpractice and the Health Toll of Western Military Interventions in the Eastern Mediterranean Region

**DOI:** 10.34172/ijhpm.9314

**Published:** 2025-10-07

**Authors:** Mohammad Karamouzian

**Affiliations:** ^1^Centre On Drug Policy Evaluation, MAP Centre for Urban Health Solutions, St. Michael’s Hospital, Toronto, ON, Canada.; ^2^Dalla Lana School of Public Health, University of Toronto, Toronto, ON, Canada.; ^3^HIV/STI Surveillance Research Center, and WHO Collaborating Center for HIV Surveillance, Institute for Futures Studies in Health, Kerman University of Medical Sciences, Kerman, Iran.

 By the end of 2024, 123.2 million people, roughly one in every 67 worldwide, were forcibly displaced, and 40% of them were children.^1^ Although mainstream media often focus on a “refugee crisis” impacting Western high-income countries, low- and middle-income countries are the ones that host 73% of the world’s refugees, 67% of whom remain in their homelands’ neighbouring countries. Indeed, only five host countries—Iran, Türkiye, Colombia, Germany, and Uganda—now shelter 37% of all displaced people in need of international protection.^2^

 The Eastern Mediterranean Region (EMR) accounts for 55% of the world’s refugees, with 33.7 million forcibly displaced people and 140 million (19% of the population) in need of humanitarian assistance.^3^ Among the 22 Member States in the region, 13 are impacted by conflicts either directly or indirectly, nine are categorized by the World Bank as “fragile or conflict-affected,” and six rank among the world’s lowest in political stability and absence of violence indicators.^3^ Indeed, extended conflicts in Afghanistan, Iraq, Palestine, Syria, and Yemen have generated the world’s largest populations of refugees and internally displaced persons (IDPs), placing extraordinary pressure on national health systems and international humanitarian efforts.^1,4^ Syria alone remains one of the world’s largest displacement crises; from an estimated population of 24 million over half have been displaced (ie, 6.1 million refugees and asylum-seekers and 7.4 million IDPs) by the end of 2024.^1^ Afghanistan (population ~43 million), Iraq (population ~46 million), and Yemen (population ~40 million) continue to generate millions of refugees and IDPs, with Afghanistan’s refugee population exceeding 6.1 million, Iraq hosting over 1 million IDPs, and Yemen having 4.7 million IDPs as of 2024.^2^ While the drivers of displacement in the EMR are multifaceted (eg, natural disasters, direct violence, persecution, economic instability, and the systematic destruction of civilian infrastructure, including health, housing, industries, and education),^3^ the context often missing from these statistics is the role of Western-led military interventions. This analytical viewpoint examines illustrative cases from EMR selected based on three criteria: (1) direct Western military involvement or substantial diplomatic/military support, (2) significant displacement outcomes (eg, >1 million displaced persons), and (3) documented health system collapse with available data from international organizations (eg, the World Health Organization [WHO], United Nations High Commissioner for Refugees). These cases—Afghanistan, Iraq, Palestine, Syria, and Yemen—represent major displacement crises where Western policy decisions coincided with systematic health infrastructure destruction.^4^ Shaped by political rhetoric that portrays military actions as humanitarian “hearts and minds” operations—often invoked in the context of the “war on terror”—and by media coverage that defaults to an “emergency imaginary,” the dominant discourse in Western capitals consistently frames these events as isolated humanitarian emergencies, disconnected from the geopolitical decisions that have caused them.^5^

 These decisions, however, are not simple policy errors; they represent a form of geopolitical malpractice. In medicine, malpractice is not merely a mistake, but a violation of the standard of care, where negligence causes foreseeable harm.^6^ Proponents of these interventions often cite humanitarian aims (eg, the responsibility to protect) or national security imperatives as justification, framing the resulting health crises as unfortunate but unavoidable collateral damage. The geopolitical malpractice framework, however, challenges this by shifting the focus from stated intent to foreseeable outcome and professional negligence. In clinical practice, a surgeon who uses a non-sterile instrument is negligent regardless of their intention to save the patient. Similarly, when foreign policy decision-makers repeatedly use interventions that predictably lead to the collapse of health systems, they are violating a fundamental standard of care, regardless of the stated geopolitical rationale. This concept offers a distinct perspective on how Western military interventions, often justified as necessary “surgical” actions to restore regional stability in EMR, have paradoxically led to the very crises of displacement and failures in health systems that we witness today. While establishing direct causation for any single conflict is complex, the evidence for malpractice rests on a consistent pattern where military interventions are repeatedly followed by health system collapse across diverse contexts, linked by a clear and predictable mechanistic pathway (Figure).

**Figure:**
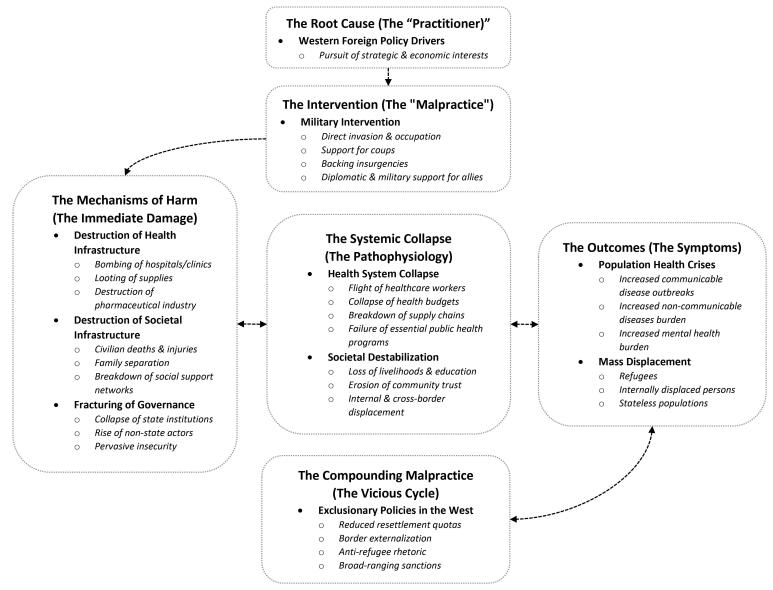
Geopolitical Malpractice in the Middle East and Its Health Consequences.

## A Chronic Pattern of Malpractice

 The health crises in the EMR are symptoms of a chronic condition, where over the past seven decades, Western foreign powers have continuously intervened in the region to achieve their strategic and economic objectives, regardless of the short- and long-term human and social costs for local populations. While a comprehensive history would include decades of proxy conflicts and covert support,^7^ this analysis focuses on key instances of direct military intervention and governance that exemplify a consistent pattern of geopolitical malpractice. The 1953 US-led coup in Iran to control Iranian oil reserves destroyed national stability by toppling a democratically elected government, which resulted in decades of authoritarian rule and intensified anti-Western sentiment that culminated in the 1979 Iranian revolution. This approach of direct intervention intensified throughout the decade. During the 1956 Suez Crisis, Britain, France, and Israel launched a military operation to retake the Suez Canal from Egypt, exposing the region’s vulnerability to external manipulation. Meanwhile, the 1958 US military intervention in Lebanon reinforced the precedent of foreign troops shaping local politics in the region. The 1963 US-supported Ba’ath Party takeover in Iraq allowed Saddam Hussein to seize power, which established a cycle of instability that persists to the present day. Collectively, these events (ie, oil-driven coups, canal crises, and troop deployments) likely normalized the use of military leverage as a “first-line intervention” of Western policies in EMR.^7,8^ Later, during the Cold War era, superpower competition between the US and the Soviet Union led to the invasion of Afghanistan in 1979-1989 and US backing of the Mujahideen insurgency, which ultimately led to the Taliban takeover and prolonged security threats.^9^ Viewed collectively, these interventions are far from being isolated incidents and constitute a clear modus operandi of geopolitical malpractice, where the prioritization of short-term strategic advantages created the very governance disruptions that established a predictable cycle of harm. This legacy of negligence was repeatedly inflicted not on stable landscapes but on regions already burdened by authoritarian governance, internal sectarian divisions, and the strategic maneuvering of rival powers. In these fragile contexts, the military interventions acted as the decisive catalyst, transforming chronic dysfunction into the acute, systemic health and humanitarian catastrophes.

## The Mechanism of Harm and Foreseeable Health Complications

 Modern military interventions weaken population health through a cascading mechanism of harm. The initial damage is multi-faceted where interventions simultaneously fracture state governance, destroy physical infrastructure, and shred the social fabric. The resulting collapse of state authority creates a vacuum, precipitating a functional paralysis of the health system itself. This secondary collapse, characterized by the flight of medical personnel, the termination of health budgets, and the breakdown of supply chains, inevitably leads to the foreseeable health complications. The 2003 US- and UK-led invasion of Iraq provides a textbook example, where the dismantling of the Ba’athist state apparatus created a profound governance vacuum, which in turn enabled the widespread and systemic insecurity that followed. The extensive looting that occurred during and after the invasion left numerous primary healthcare centers without power or access to clean water. The security situation forced half of Iraq’s 18 000 doctors to leave the country by 2007, while essential hospital equipment disappeared, and 2000 doctors were killed during this period.^10^ The Syrian civil war further exposed healthcare facilities to deliberate attacks that showed how health systems could easily become tools for combat.^10^ Public hospitals in Syria suffered damage to the point where more than half were either damaged or destroyed, and a pharmaceutical industry that used to supply neighbouring regions was destroyed, leading to chronic shortages of essential medications.^11^ More recently, public health infrastructure in Afghanistan almost collapsed in 2021 after US troops abruptly withdrew and terminated the international funding assistance, responsible for 75% of the health budget, affecting over 2 million people.^3,12^

 This pattern of health system destruction is not simply historical; it is ongoing, as observed in the ongoing wars in Gaza and Yemen. In Gaza, Israeli military operations, reinforced by strong US military and diplomatic support, including repeated vetoes of Security Council calls for a permanent ceasefire,^13^ have resulted in the near-total collapse of the healthcare system.^3^ The WHO has documented 28 attacks on Gaza’s healthcare infrastructure since October 2023, killing over 1580 health workers, leaving only half (19 out of 36) of its hospitals partially functional by May 2025, with a total of only 2000 beds available for a population of 2.1 million.^14,15^ This systemic dismantling extends beyond healthcare, destroying 92% of homes, 83% of croplands, 83% of water wells, 71% of greenhouses, and 72% of the fishing fleet.^15^ The population-level harms have also been catastrophic, with over 1.9 million people internally displaced, and more than 65 000 people, including over 18 400 children and 9700 women, killed in the past two years.^15^

 The war in Yemen, fueled by a Saudi-led coalition with significant military and logistical support from the US, UK, and France, provides another blunt image of this destructive mechanism. The conflict escalated into a brutal deadlock after the coalition’s 2015 intervention, which aimed to restore the internationally recognized Yemeni government following the Houthi movement’s seizure of the capital, Sanaa. Ten years of conflict have triggered a near-total institutional collapse. Only half of Yemen’s health facilities remain fully operational, crippled by a lack of medicines, fuel, and equipment, while most public health workers have gone without regular salaries for years.^3,16^ This health crisis is embedded within a broader societal collapse that has shrunk the economy by half and pushed over 80% of the population below the poverty line. Consequently, the population-level harms are immense, with over 18 million requiring humanitarian assistance, and 17 million facing food insecurity.^17^ This humanitarian crisis has been further compounded by direct recent US attacks on Houthi targets in March 2025, and their re-designation as a terrorist organization in January 2024; interventions that have created significant barriers to the delivery of international aid, exacerbating the resurgence of preventable diseases like measles, diphtheria, and polio.^3,18^

 These systemic collapses predictably manifest as a spectrum of acute population health crises, the clinical symptoms of a society in critical condition. The Syrian population, for instance, experienced a decline in life expectancy from 73.5 years in 2010 to 63.2 years in 2015,^19^ while in Gaza, about two million people are facing extreme food insecurity.^15^ In Afghanistan, the collapse of health services left nearly two-thirds of the population requiring humanitarian assistance by 2023.^12^ The breakdown of immunization programs creates particularly ideal conditions for the spread of communicable diseases; in Iraq, the drop in immunization rates from 60.7% in 2000 to 38.5% in 2006 directly contributed to recurrent polio and cholera outbreaks,^20^ while a similar collapse of services in Syria led to the 2013-2014 polio outbreak that infected 36 children.^21^ In Yemen, the breakdown of public services is also reflected in the resurgence of preventable diseases, as the rate of unvaccinated children has reached 28%.^3^ Non-communicable diseases pose another substantial health burden to displaced populations in the EMR. Refugees from Syria who are >30 years old and reside in northern Jordan have an estimated 39.5% hypertension and 19.3% diabetes.^22^ These medical conditions, usually manageable, transform into fatal threats when supply chains break down and healthcare remains inaccessible in the overburdened healthcare systems of neighbouring host countries.^21^ Mental health outcomes are equally dire. While comprehensive data for all displaced persons in the region are limited, a systematic review on women in the EMR’s fragile settings reveals a crisis of immense scale^23^; prevalence estimates for post-traumatic stress disorder (52%) and depression (43%) far exceed the already high global pooled estimates for refugees (31.4% and 31.5%, respectively).^24^ This trauma stems not only from direct violence but from observing the collapse of the social and health institutions that once provided stability.

## The Stark Paradox of Exclusionary and Punitive Policies

 The harm of this geopolitical malpractice is compounded when systemic negligence escalates into a refusal to address the subsequent consequences. The same Western actors who are responsible for causing the initial health crisis then implement policies that actively block the remedy for displaced populations. The US demonstrates this pattern through its actions, spending $5.8 trillion on wars in the EMR in the past two decades that led to millions of displaced people,^25^ while reducing refugee resettlement quotas from 90 000 in 2000 to 18 000 in 2020.^26^ The 2017 “Muslim ban” excluded refugees from countries affected by US military interventions, thus effectively severing responsibility for those whose displacement it helped cause.^27^ The European Union (EU) demonstrates similar policy malpractice through the externalization of its borders. The 2016 EU-Türkiye deal and partnerships with Libya outsource migration control to countries with documented human rights abuses, where refugees face violence and denial of medical care.^28^ This creates a secondary layer of predictable harm; refugees fleeing conflicts partly caused by Western interventions are then subjected to additional trauma in detention centers funded by those same Western powers. This pattern, however, extends beyond migration policy to include economic pressure. Broad-ranging sanctions imposed by both the US and the EU on several EMR countries (eg, Afghanistan, Iran, Iraq, Lebanon, Syria, and Yemen), regardless of their stated geopolitical aims, have the practical effect of crippling national economies and impeding access to essential medicines and medical equipment.^29^ This serves to further degrade health systems already weakened by conflict, effectively punishing the civilian population and obstructing any path to recovery.

## Toward a Standard of Care for Foreign Policy

 Just as medicine developed standards of care to prevent malpractice, Western foreign policy requires evidence-based safeguards to ensure accountability. Accountability must operate on four levels of domestic oversight, reparative finance, legal deterrence, and shared human responsibility. First, accountability must begin before an intervention is launched. Before governments (eg, the US or EU), can authorize military force abroad, their own legislative bodies must require and publicly debate a comparative policy health analysis to independently evaluate the foreseeable health and stability consequences across a range of policy options, from sustained diplomacy and targeted sanctions to military intervention. Such a process forces a transparent evaluation of trade-offs, shifting health from a post-hoc damage assessment to an upfront strategic consideration and creating a concrete tool for democratic oversight. Second, accountability requires reparative justice for the harm caused. The international community should establish a fund based on the “Polluter Pays Principle,”^30^ requiring countries leading interventions outside of a direct UN mandate to make legally-binding contributions toward rebuilding the health infrastructure they damage. This fund, potentially administered by a neutral body like the World Bank or a dedicated United Nations agency, would help institutionalize the principle that destroying public health infrastructure carries a tangible, non-negotiable cost. Third, legal accountability is essential. The global health community must advocate for the consistent application of international humanitarian law, pressuring countries to accept the International Criminal Court’s jurisdiction over the actions of their own military forces, a step many powerful countries currently resist. The practical role for the health community is to continuously highlight this double standard, framing non-compliance not just as a legal issue, but as a public health imperative. Lastly, true accountability extends to the human consequences. Countries whose interventions have been a primary driver of displacement have a special moral and financial responsibility to support refugee populations, both by robustly funding host nations and by expanding their own resettlement programs and streamlining credential recognition for refugee healthcare workers. In the context of rising anti-refugee sentiment, it is essential to reframe refugee support not as an act of charity, but as a fundamental duty of reparative justice.

 The evidence is clear. Western interventions in the EMR have contributed to a predictable pattern of harm, systematically destroying health systems and generating displaced populations with preventable health crises. These results stem directly from policy decisions where geopolitical and economic interests were pursued without adequate consideration for the foreseeable human costs. The medical profession’s response to malpractice—developing standards of care, ensuring accountability, and embracing the principle of “first, do no harm”—provides a roadmap for Western foreign policy reform in the EMR. The international health policy community must demand the same standards from foreign policy that we expect from medical practice. Anything less cements the cycle of instability and preventable suffering for the foreseeable future in EMR and beyond.

## Ethical issues

 Not applicable.

## Conflicts of interest

 Author declares that he has no conflicts of interest.
